# Plant growth-promoting rhizobacterium *Pseudomonas* PS01 induces salt tolerance in *Arabidopsis thaliana*

**DOI:** 10.1186/s13104-019-4046-1

**Published:** 2019-01-11

**Authors:** Thanh Nguyen Chu, Bao Thi Hoai Tran, Le Van Bui, Minh Thi Thanh Hoang

**Affiliations:** Department of Plant Biotechnology and Biotransformation, Faculty of Biology and Biotechnology, University of Science, Vietnam National University-Ho Chi Minh City, Ho Chi Minh City, Vietnam

**Keywords:** Arabidopsis *thaliana*, Plant growth-promoting rhizobacteria (PGPR), *Pseudomonas* PS01, Salt stress tolerance

## Abstract

**Objectives:**

Plant growth-promoting rhizobacteria (PGPR) may contribute to sustainable crop production by improving plant growth and/or plant tolerance to abiotic stresses. Soil salinity, which limits the productivity of crop plants, is one of the major concerns of modern agriculture, especially in countries heavily affected by climate change as Vietnam. Currently, only a few reports have studied local PGPR isolated in Vietnam, particular *Pseudomonas*. Therefore, our study aimed to isolate and identify a region-specific *Pseudomonas* strain and evaluate the effects of this strain on germination, growth promotion and gene expression of *Arabidopsis thaliana* under salt stress.

**Results:**

The *Pseudomonas* named PS01 was isolated from maize rhizosphere collected in Ben Tre province, Vietnam. This strain was identified as a member of the *Pseudomonas putida* subclade. *Pseudomonas* PS01 could improve the germination rate of *Arabidopsis* seeds in 150 mM NaCl. *A. thaliana* plants inoculated with *Pseudomonas* PS01 survived under salt stress conditions up to 225 mM NaCl, while all non-inoculated plants were dead above 200 mM NaCl. The transcriptional levels of genes related to stress tolerance showed that only *LOX2* was up-regulated, while *APX2* and *GLYI7* were down-regulated in inoculated plants in comparison to the non-inoculated controls. In turn, *RD29A* and *RD29B* did not show any significant changes in their expression profiles.

**Electronic supplementary material:**

The online version of this article (10.1186/s13104-019-4046-1) contains supplementary material, which is available to authorized users.

## Introduction

Soil salinity is a widespread problem that limits crop yield and cultivation throughout the world, including the Mekong Delta, Vietnam [1]. Salinity creates ion imbalance and generates highly reactive oxygen species (ROS) in plants, which causes ion toxicity and oxidative stress [2, 3]. This, in turn, leads to plant growth inhibition, slower development, senescence and death. To improve plant salinity tolerance, several strategies, such as the use of fertilizers, traditional breeding and genetic engineering, have been extensively studied for decades [4]. The application of plant growth-promoting rhizobacteria (PGPR) is one of the most promising alternative approaches to improve crop production in saline soils [2, 4, 5]. Various salt-tolerant PGPR including *Azospirillum, Burkholderia, Rhizobium*, *Pseudomonas*, *Acetobacter* and *Bacillus* have been successfully applied or tested for plant growth promotion under salt stress [6, 7]. The fluorescent *Pseudomonas* is considered an important model to assess beneficial plant–bacteria interactions, including plant growth promotion under abiotic stress [8, 9]. Inoculation of plants with *Pseudomonas* was found to alleviate salinity effects on plant development by reducing the uptake of toxic ions, inducing systemic resistance, producing phytohormones, increasing nutrient uptake and establishing root colonization [10–14]. *Pseudomonas*-induced salt tolerance has been mainly studied at the physiological and biochemical levels in plants. However, little is known about the transcriptional changes of plant salt-responsive genes in an interactive process [14].

To cope with salt stress, early plant responses include synthesis of ROS scavengers, detoxification of ROS and abscisic acid (ABA) signaling [15–17]. The glyoxalase pathway, which degrades methylglyoxal, is one of the main detoxification pathways [18]. In the ABA response, the expression of *RD29* (Responsive to Desiccation) genes including *RD29A* and *RD29B* is induced by salt stress [19]. PGPR may significantly enhance plant antioxidant activities, and thus protect plants from salt toxicity, by increasing the expression of enzymes such as ascorbate peroxidase (APX), superoxide dismutase (SOD) and catalase [16, 19, 20]. Interestingly, *A. thaliana* inoculated with PGPR such as *Burkholderia phytofirmans* PsJN and *Enterobacter* spp. EJ01 showed an enhanced tolerance to salt stress that involved transcriptional changes of genes related to early stress responses [16, 19].

It is necessary to identify native microbial strains which can be used in regional crops as potential plant growth promoters to achieve desirable yields [21]. The application of indigenous PGPR will provide more advantages for regional crops since PGPR can easily acclimatize to the local environmental conditions and enhance the plant–microbe interactions [22]. In previous study, we successfully isolated and identified some *Pseudomonas* spp. capable of enhancing plant growth in Vietnam [23]. Therefore, in this study, we aimed to isolate a *Pseudomonas* strain that can increase salt stress tolerance and to investigate the underlying molecular mechanisms.

## Main text

### Materials and methods

#### Bacterial isolation

Rhizobacteria were isolated from maize (*Zea mays* L.) rhizosphere collected in Ben Tre province, Vietnam. A soil suspension was obtained by shaking roots with adhered soil in phosphate-buffered saline for 10 min at 180 rpm. The suspension was serially diluted, spread onto King’B medium (KB) [24] and incubated at 30 °C for 24 h. A single colony was picked up and re-cultured a few times on solid KB to obtain a pure culture. The presence of fluorescent *Pseudomonas* was examined under UV light. Total isolates were grown on different concentration NaCl (0–10%) to evaluate salt tolerance property.

#### Bacterial identification

For phenotyping, the bacterial strain was identified according to morphological and chemotaxonomic characters based on the Bergey’s Manual of Determinative Bacteriology. For genotyping, bacterial genomic DNA was extracted and purified using the Wizard Genomic DNA purification kit (Promega, USA). The complete 16S rDNA was amplified by using PCR with the universal bacterial primers 27F and 1492R (Additional file 1: Table S1) [25]. The PCR product was sequenced and analyzed with BLASTn to identify the strain genus. Simultaneously, a fragment of *rpoD* gene was amplified by using the primers rpoD 70F and rpoD 70R (Additional file 1: Table S1) [26, 27]. The sequences of related species and genera were obtained from GenBank. Multiple sequence alignments were performed by ClustalX; the phylogenetic analysis was determined by employing the neighbor-joining method. The phylogenetic tree was constructed with MEGA version 6 Software [28].

#### Plant growth conditions and treatments

To define whether the bacteria had an effect on the germination of *Arabidopsis* in normal and saline condition, sterilized and synchronized seeds were inoculated with bacterial suspension (10^6^ CFU/ml), or MgCl_2_ solution as a control or nongrowth-promoting strain *Escherichia coli* DH5α as a negative control, and germinated on solid, half-strength Murashige and Skoog medium (MS ½) plates with or without 150 mM NaCl. Plates were placed in a growth chamber at 22 °C with a photoperiod of 16/8 h (light/dark). Four days after sowing (DAS), the seed germination percentage was determined.

To investigate the effect of *Pseudomonas* PS01 on *Arabidopsis* salt tolerance under different NaCl concentrations, 4-day-old seedlings were transferred to solid MS ½ supplemented with different concentrations of this salt. Plates were placed vertically in a growth chamber at 22 °C with a photoperiod of 16/8 h (light/dark). After 7 days, the plant survival rate was determined.

#### RNA extraction and real-time PCR (RT-PCR) analyses

RNA extraction was performed on plantlets after being transplanted to MS ½ alone or supplemented with 150 mM NaCl for 24 h. RNA was obtained by using the Trizol (Invitrogen™, USA) method. RT-PCR was performed by using the Luna Universal One-Step RT-PCR Kit (New England Biolabs, USA). PCR primers are shown in Additional file 2: Table S2. The relative transcript level (RTL) was calculated by normalizing to *ACT2* as follows: RTL = 2^∆∆Ct^, where ∆∆Ct = ∆Ct(gene) − ∆Ct(*ACT2*). All experiments were performed with three biological and two technical replicates.

#### Statistical analysis

For comparison between treatments, ANOVA was performed with Graphpad Prism 7.0.

### Results

#### Identification of bacteria

**Fig. 1:**
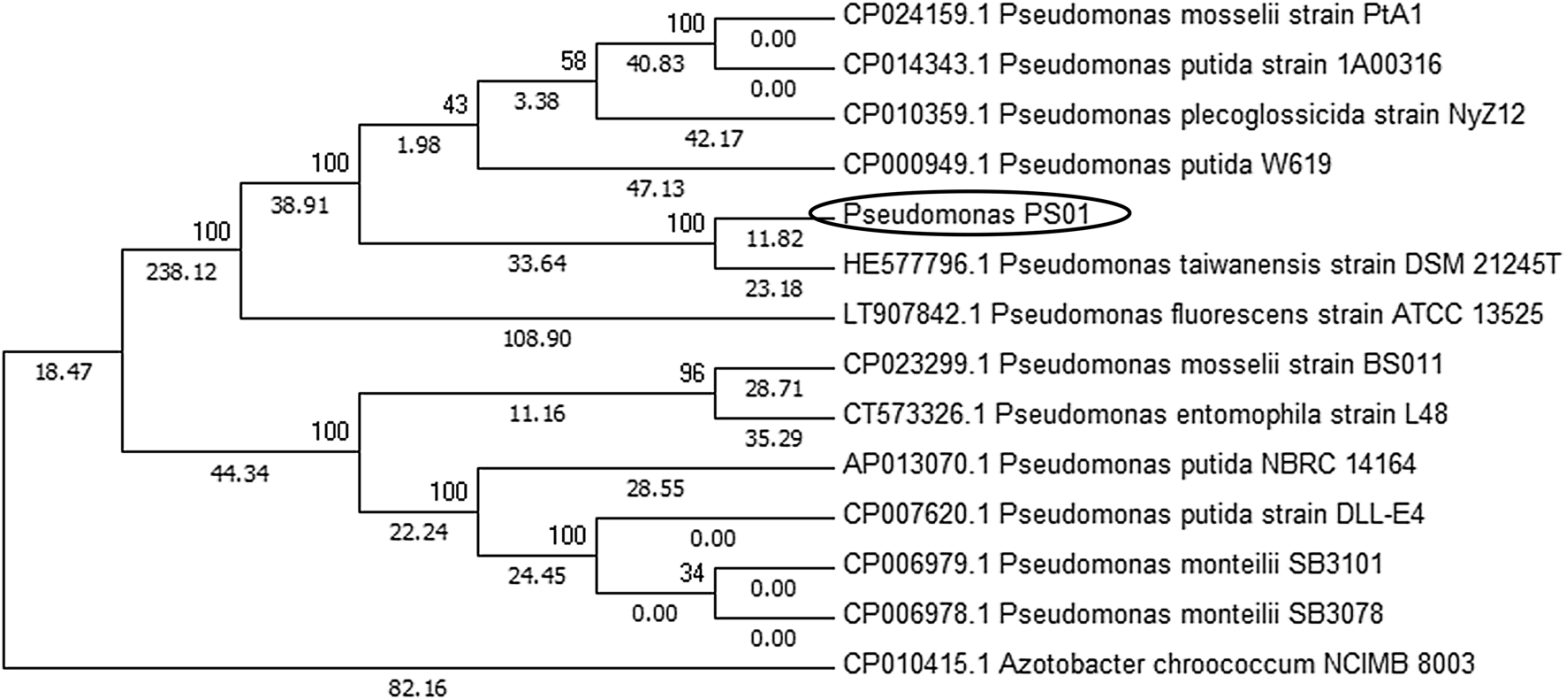
Phylogenetic tree based on *rpoD* gene sequences of *Pseudomonas* PS01, related *Pseudomonas* strains and *Azotobacter chroococcum*. The sequence of *Pseudomonas* PS01 showed 99% similarity to *Pseudomonas taiwanesis* strain (GenBank accession number HE577796.1). The bootstrap values are inferred from 1000 replicates. Branch lengths are presented to phylogenetic distances

Seventeen rhizobacterial strains were isolated from maize rhizosphere. The salt tolerant properties results revealed that out of 17 bacterial isolates tested; only isolate named PS01 was able to grow in the presence of 8% NaCl (Additional file 3: Fig. S1). Based on its growth curve, PS01 strain has an optimal growth temperature of 30 °C. Morphological and chemotaxonomic analyses revealed that PS01 is rod-shaped, Gram-negative, aerobic, non-spore-forming, catalase-positive and oxidase-positive, and fluoresces under UV light at 365 nm (Additional file 4: Fig. S2). The BLAST search of 16S rDNA against the GenBank indicated that PS01 is most similar to *Pseudomonas* spp. In this genus, the *rpoD* gene has been identified as one of the best biomarkers for gene phylogeny, which correlates well with that of the 16S rRNA gene [26, 27, 29, 30]. Therefore, *rpoD* was used to identify PS01 in our study. The phylogenetic tree of *rpoD* gene indicated that PS01 belongs to the *Pseudomonas putida* subclade (Fig. 1).

#### *Pseudomonas* PS01 enhances seed germination rate in salt stress conditions

**Fig. 2:**
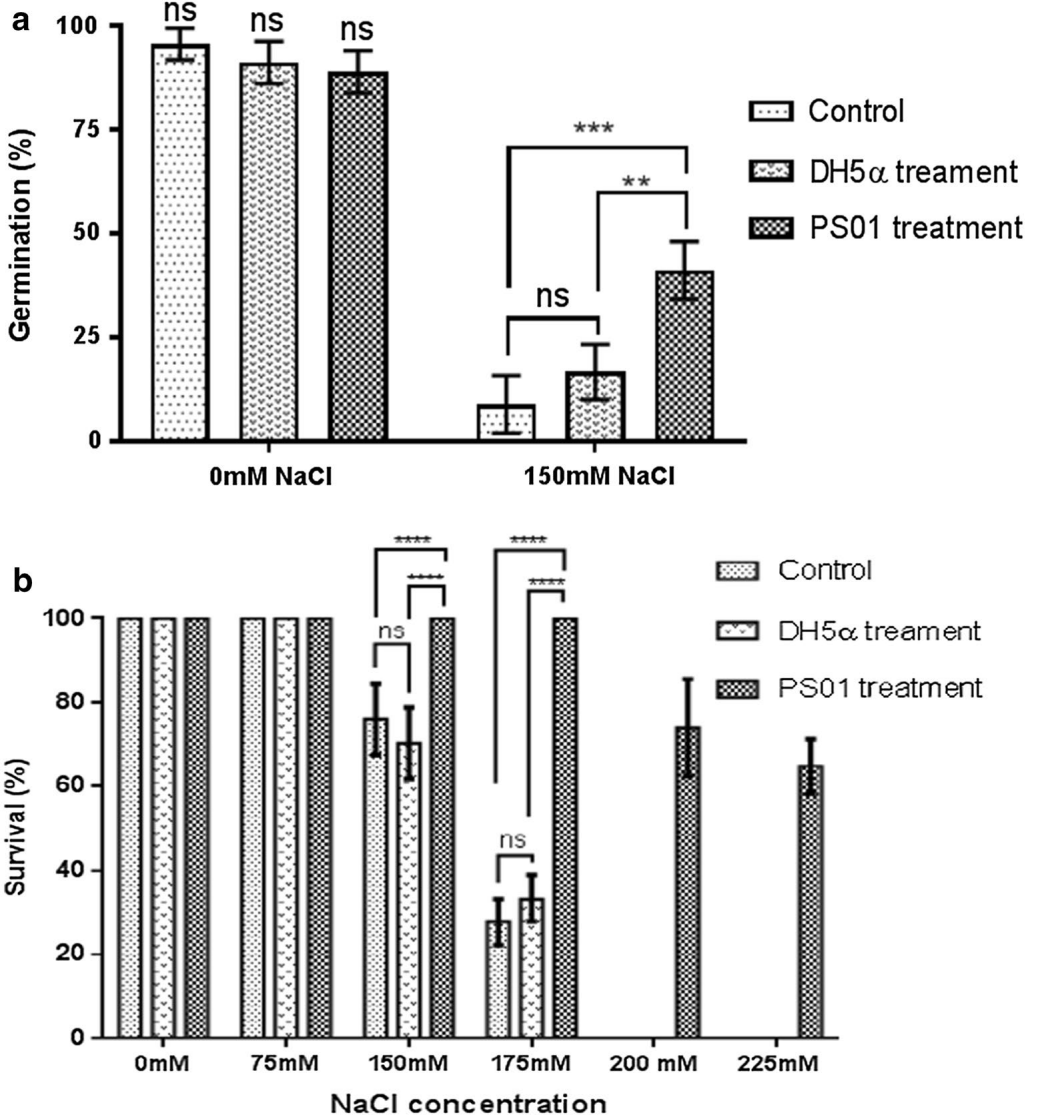
*Pseudomonas* PS01 enhances *Arabidopsis thaliana* germination and survival under high saline concentration. Germination rate of non-inoculated, inoculated DH5α and inoculated PS01 *Arabidopsis* seeds in MS ½ medium alone and with 150 mM NaCl added (**a**). Survival rate (%) of non-inoculated, DH5α-inoculated and PS01-inoculated plants grown on medium supplemented with different NaCl concentrations (**b**). Data are represented as mean ± SE of at least 30 seeds or plants per treatment. Experiments were repeated at least three times with similar results. **, ***, ****Indicates the significant difference between both treatments (control vs PS01-inoculated) at p value < 0.01, < 0.001 and p < 0.0001, respectively

To test whether PS01 could enhance the germination rate of *A. thaliana*, its effect was examined in MS ½ media with or without 150 mM NaCl. We observed that the germination rate in the 150 mM NaCl treatment was significantly increased in PS01-inoculated seeds when compared to the control. PS01-treated *A. thaliana* seeds showed 30.7% germination rate, while this value was only 9.5% in the control (Fig. 2a). However, no significant difference in the seed germination rate could be observed between the inoculated and non-inoculated seeds in MS ½, suggesting that the PS01 treatment has no effect on *A. thaliana* germination in normal conditions.

#### *Pseudomonas* PS01 enhances salt stress tolerance

To address the effects of NaCl and PS01 on *A. thaliana* salt tolerance in vitro, the survival rates (%) of PS01-inoculated and non-inoculated plants grown on media supplemented with NaCl concentrations ranging from 75 mM to 225 mM were evaluated. On the 7th day after being transferred to the salt stress media, *Arabidopsis* seedlings were observed and photographed to identify the number of surviving plants.

PS01 root colonization was shown to increase the survival of plants exposed to saline concentrations ranging from 75 to 225 mM NaCl (Additional file 5: Fig. S3). For instance, all plants inoculated with PS01 could survive the 175 mM NaCl treatment as opposed to only 30–40% of the controls (Fig. 2b). These results suggest that *Pseudomonas* PS01 may enhance *A. thaliana* survival under salt stress.

#### *Pseudomonas* PS01 induces transcriptional changes in salt-stressed *A. thaliana* plants

To investigate the molecular mechanisms of PS01-induced salt stress tolerance in *Arabidopsis*, some genes related to early salt stress responses such as ROS scavenging (*APX2*), detoxification (*GLYI7*), ABA signaling (*RD29A* and *RD29B*) and JA synthesis (*LOX2*) were chosen for quantitative RT-PCR analysis. The gene expression profiles of these genes were obtained from 4 different samples: seedlings inoculated or non-inoculated with PS01 and grown in MS ½ alone or transferred to MS ½ supplemented with 150 mM NaCl.

**Fig. 3:**
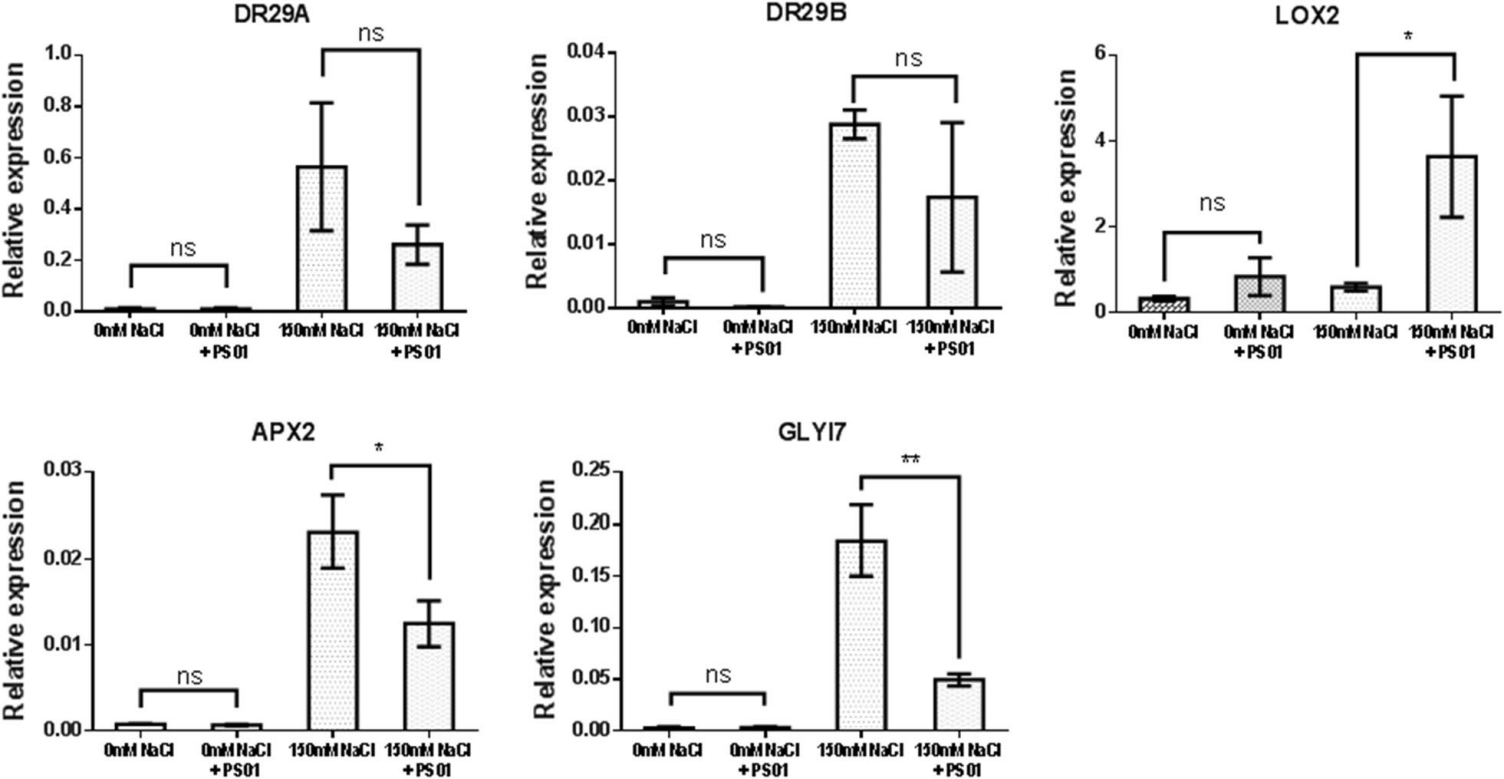
Gene expression analysis of abiotic stress-responsive genes of *Arabidopsis* seedlings inoculated with *Pseudomonas* PS01 after 24 h of being transferred to salt stress conditions. The transcriptional levels of *RD29A*, *RD29B*, *LOX2*, *APX2* and *GLYI7* of PS01-inoculated and non-inoculated *A. thaliana* seedlings grown in MS ½ alone or transferred to MS ½ supplemented with 150 mM NaCl are represented. Experiment was performed with three biological and two technical replicates (*) and (**) indicate the significant difference between both treatments (150 mM NaCl vs 150 mM NaCl + PS01 inoculated) at p value < 0.05 and p < 0.01, respectively

After 24 h salt stress, the transcriptional expression of all five genes was remarkably up-regulated compared to the control (seedlings grown on MS ½ alone). The analyses showed no differences in *RD29A* and *RD29B* expression in PS01-inoculated and non-inoculated seedlings under the NaCl treatment. By contrast, *LOX2* expression was up-regulated in PS01-inoculated, salt-stressed plants, while *APX2* and *GLYI7* were significantly down-regulated (Fig. 3).

### Discussion

PS01 is the first *Pseudomonas* strain isolated in Vietnam which alleviates the effect of salinity on plant growth. PS01 belongs to the *Pseudomonas putida* subclade based on the *rpoD* gene tree (Fig. 1), being closely related to *P. taiwanensis* (GenBank accession number HE577796.1). Interestingly, *P. putida* was already known to reduce the detrimental effect of salinity on germination and plant growth [7, 31].

In this study, *Pseudomonas* PS01 improved the germination rate of *A. thaliana* on salt stress medium (MS ½ supplemented with 150 mM NaCl). Compared to previous studies focusing on the effect of bacteria on plant growth, the germination process was accelerated and its period was also extended [32]. For example, *P. putida* R4 and *P. chlororaphis* R5 improved cotton seed germination in response to NaCl stress up to 64 and 73%, respectively [7]. In contrast, *Pseudomonas* spp. PDMZnCd2003 negatively affected rice germination, and reduced seedling shoot and root length [33]. We have shown that PS01 helps improve salt stress tolerance in *A. thaliana* seedlings. Under salt stress conditions, the transcriptional level increase of *GLYI7* and *APX2* in seedlings inoculated with PS01 was lower than that of non-inoculated seedlings. Glyoxalase can be considered as an alarm in the abiotic stress response in plants [34]. In turn, ascorbate peroxidases catalyze the conversion of H_2_O_2_ into H_2_O. The down-regulation of *GLYI7* and *APX2* suggests that inoculation with PS01 may reduce stress level in plants. Therefore, the mechanisms contributing to the survival of inoculated plants during salt stress can be related to other molecular pathways. Alternatively, the production of biofilms by the bacteria could be beneficial to plant survival under stress conditions as shown in recent reports, in which bacterial exopolysaccharide (EPS) and biofilm formation stimulated plant growth under salt stress by reducing Na^+^ uptake by the plant [20, 35]. However, further evidences for EPS production or other putative mechanisms participating in the PS01-mediated salt tolerance of plants need to be verified.

Jasmonate (JA) is a positive regulator of the salt stress response that accumulates rapidly when plants are submitted to stress [20, 36]. *LOX2* encodes a lipoxygenase that constitutes an essential component of the JA synthesis pathway [37]. Compared to non-inoculated seedlings in response to salt stress, the increase of *LOX2* expression in PS01 inoculated seedlings in our study is in agreement with Cho et al. [14] and Poupin et al. [38], who also reported that PGPR such as *Pseudomonas chlororaphis O6* and *Burkholderia phytofirmans* PsJN mediated systemic resistance against abiotic stress by increasing the expression of defense genes regulated by the JA pathway [14, 38]. The phenotypic changes, transcriptional profile and spatiotemporal responses under salt stress of plants inoculated with PS01 will be analyzed at different plant growth stages to clarify this pathway.

### Conclusion

Our study has shown that *Pseudomonas* PS01 can improve *Arabidopsis thaliana* germination and survival rate under salt stress.

## Limitations

Although, mechanism underlying PS01-Arabidopsis interaction to enhance plant salt tolerance has not been fully discovered, further studies have been conducting in our group to provide new insights into this interaction including whole genome analysis and transposon mutant library screening of PS01, as well as long-term transcriptomic analysis of plants inoculated with this isolate. PS01 will be inoculated with maize under salt stress and be developed as a biofertilizers in the future prospect.

## Additional files

**Additional file 1: Table S1.** Primers used to amplify 16S DNA and *rpoD* gene.

**Additional file 2: Table S2.** List of RT-PCR primers used in this study.

**Additional file 3: Figure S1.** Growth of *Pseudomonas* PS01 on TSB medium at different NaCl concentrations: 0% (A), 2% (B), 4% (C), 6% (D), 8% (E) and 10% (F).

**Additional file 4: Figure S2.** *Pseudomonas* PS01 colonies on King’s B medium (A) and visualization of fluorescent colonies under UV light (B). Picture B was taken using 365 nm as excitation wavelength. Gram staining of *Pseudomonas* PS01 cells (C).

**Additional file 5: Figure S3.** Effects of NaCl and PS01 on *A. thaliana* salt tolerance in vitro under different NaCl concentrations. Non-inoculated *A. thaliana* grown on MS ½ supplemented with NaCl 0 mM (A1), NaCl 75 mM (A2), NaCl 150 mM (A3),175 mM (A4), 200 mM (A5), 225 mM (A6). *A. thaliana* inoculated with *E. coli* grown on MS ½ supplemented with NaCl 0 mM (B1), NaCl 75 mM (B2), NaCl 150 mM (B3), 175 mM (B4), 200 mM (B5), 225 mM (B6). *A. thaliana* inoculated with PS01 grown on MS ½ supplemented with NaCl 0 mM (C1), NaCl 75 mM (C2), NaCl 150 mM (C3), 175 mM (C4), 200 mM (C5), 225 mM (C6). White bars in the photographs correspond to 1 cm.

## Abbreviations

ABA: abscisicacid
APX: ascorbateperoxidase
*A. thaliana*: *Arabidopsis thaliana*
DAS: days after sowing
EPS: exopolysaccharide
*GLYI7*: *Glyoxalase I 7*
JA: Jasmonate
KB: King’B medium
*LOX2*: *Lipoxygenase 2*
MS ½: half-strength Murashige and Skoog medium
PGPR: plantgrowth-promoting rhizobacteria
PS01: *Pseudomonas* PS01
RT-PCR: real-time PCR
*RD29A*: *Relative to Dessication A*
*RD29B*: *Relative to Dessication B*
ROS: reactive oxygen species
RTL: relative transcript level
SOD: superoxide dismutase
