# Supplementary material for: Plant growth-promoting rhizobacterium Pseudomonas PS01 induces salt tolerance in Arabidopsis thaliana
Source: BMC Res Notes. 2019 Jan 11;12:11. doi: 10.1186/s13104-019-4046-1 (PMC6330407; doi:10.1186/s13104-019-4046-1)
Supplement: Supplementary file 1 — Additional file 1: Table S1. Primers used to amplify 16S DNA and rpoD gene. [file 13104_2019_4046_MOESM1_ESM.docx]

**Additional file 1: Table S1.**

| **Target** | **Primer** | **Sequence (5’-3’)** | **Reference** |
| --- | --- | --- | --- |
| **16SDNA** | 27F | AGA GTT TGA TCC TGG CTC AG | Lane (1991) |
|  | 1492R | ACG GCT ACC TTG TTA CGA CTT |  |
| ***rpo****D* | 70F | ACG ACT GAC CCG GTA CGC ATG TAY ATG MGN GAR ATG GGN ACN GT | Yamamoto and Harayama (1998) |
|  | 70R | ATA GAA ATA ACC AGA CGT AAG TTN GCY TCN ACC ATY TCY TTY TT |  |

Lane DJ. 16S/23S rRNA sequencing. In: Stackebrandt E, Goodfellow M, editors. Nucleic acid techniques in bacterial systematics. New York, NY: John Wiley and Sons; 1991. p. 115-75. 1991:115-175.

Yamamoto S, Harayama S. Phylogenetic relationships of *Pseudornonas putida* strains deduced from the nucleotide sequences of gyrB, rpoD and 16s rRNA genes. *Int J Syst Bacteriol*. 1998; 488(1):3-8.
