# Supplementary material for: Plant growth-promoting rhizobacterium Pseudomonas PS01 induces salt tolerance in Arabidopsis thaliana
Source: BMC Res Notes. 2019 Jan 11;12:11. doi: 10.1186/s13104-019-4046-1 (PMC6330407; doi:10.1186/s13104-019-4046-1)
Supplement: Supplementary file 2 — Additional file 2: Table S2. List of RT-PCR primers used in this study. [file 13104_2019_4046_MOESM2_ESM.docx]

**Additional file 2: Table S2.**

| **Gene** | **Locus** | **Forward (5’-3’)** | **Reverse (5’-3’)** | **Tm** | **Reference** |
| --- | --- | --- | --- | --- | --- |
| *RD29A* | At5g52310 | ATCACTTGGCTCCACTGTTGTTC | ACAAAACACACATAAACATCCAAAGT | 57.0 | Bu *et al*., 2009 |
| *RD29B* | At5g52300 | GGAGTGAAGGAGACGCAACAAG | GGAATCCGAAAACCCCATAGTC | 58 | Pinedo *et al.,* 2016 |
| *APX2* | At3g09640 | TGGTCGGATGGGACTCAAT | AAGAGCCTTGTCGGTTGGT | 58.5 | Xie *et al*., 2012 |
| *LOX2* | At3g45140 | ATCAACGCTCGTGCACGCCA | CCGCGGGTAAGCCTTCCTGG | 64 | Poupin et al., 2013 |
| *GLYI7* | At1g80160 | CTACGATCGAGCCAGCGTTC | CCATGTCCAAACAACCACGC | 57.5 | Pinedo *et al.,* 2016 |

Bu Q, Li H, Zhao Q, Jiang H, Zhai Q, Zhang J, Wu X, Sun J, Xie Q, Wang D, et al. 2009. The Arabidopsis RING Finger E3 Ligase RHA2a Is a Novel Positive Regulator of Abscisic Acid Signaling during Seed Germination and Early Seedling Development. *Plant Physiol*150(1): 463-481.

Pinedo I, Ledger T, Greve M, Poupin MJ. *Burkholderia phytofirmans* PsJN induces long-term metabolic and transcriptional changes involved in *Arabidopsis thaliana* salt tolerance. *Front Plant Sci*. 2015;6(June):1-17. doi:10.3389/fpls.2015.00466.

Poupin, M.J., Timmermann, T., Vega, A., Zuniga, A., and Gonzalez, B. (2013). Effects of the plant growth-promoting bacterium *Burkholderia phytofirmans* PsJN throughout the life cycle of *Arabidopsis thal*iana. *PLoS One* 8, e69435. doi: 10.1371/journal.pone.0069435.

Xie Y, Mao Y, Lai D, Zhang W, Shen W. 2012. H_2_ Enhances *Arabidopsi*s Salt Tolerance by Manipulating ZAT10/12-Mediated Antioxidant Defence and Controlling Sodium Exclusion. *PLoS One*7(11): e49800.
