# Supplementary figures and images for: Plant growth-promoting rhizobacterium Pseudomonas PS01 induces salt tolerance in Arabidopsis thaliana
Source: BMC Res Notes. 2019 Jan 11;12:11. doi: 10.1186/s13104-019-4046-1 (PMC6330407; doi:10.1186/s13104-019-4046-1)

**Additional file 3:**


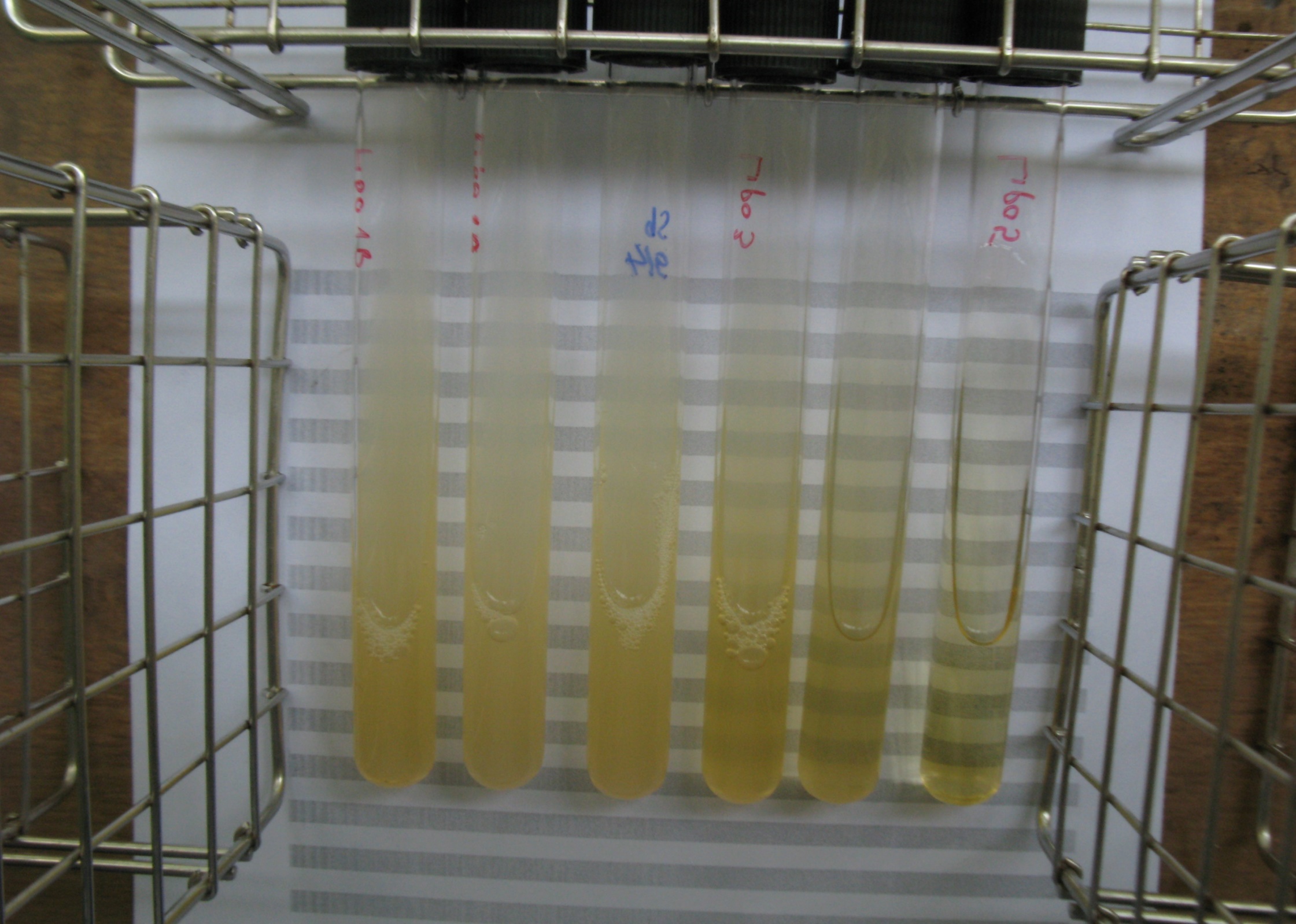


**A**

**B**

**C**

**D**

**E**

**F**

**Figure S1.**

Supplement: Supplementary file 3 — Additional file 3: Figure S1. Growth of Pseudomonas PS01 on TSB medium at different NaCl concentrations: 0% (A), 2% (B), 4% (C), 6% (D), 8% (E) and 10% (F). [file 13104_2019_4046_MOESM3_ESM.docx]

**Additional file 4:**

**
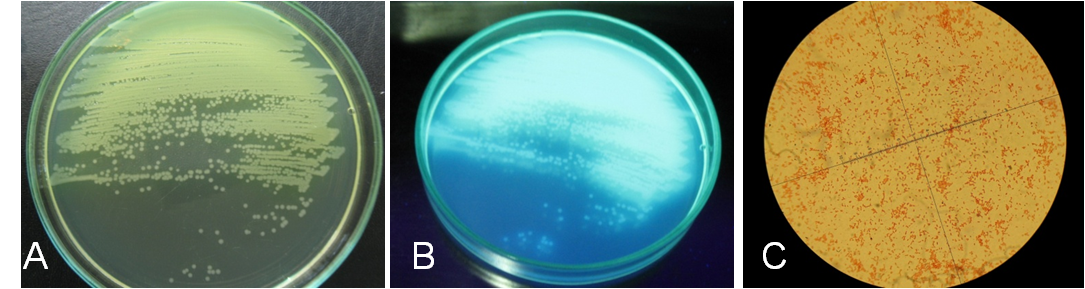
**

Supplement: Supplementary file 4 — Additional file 4: Figure S2. Pseudomonas PS01 colonies on King’s B medium (A) and visualization of fluorescent colonies under UV light (B). Picture B was taken using 365 nm as excitation wavelength. Gram staining of Pseudomonas PS01 cells (C). [file 13104_2019_4046_MOESM4_ESM.docx]

**Additional file 5:**


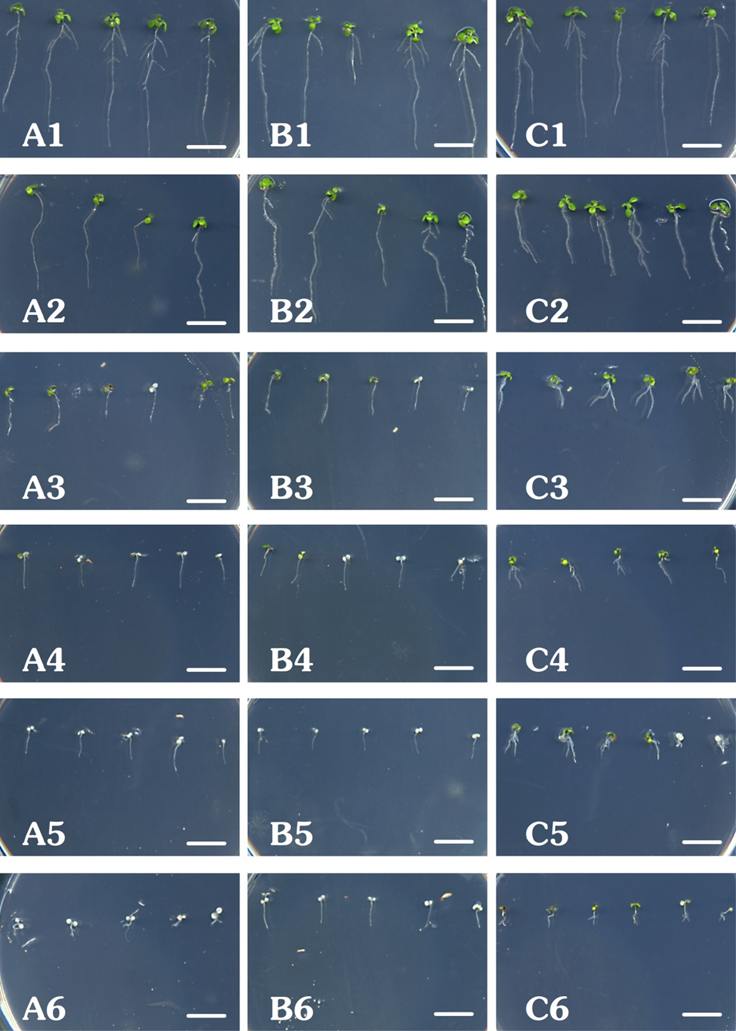


**Figure S3.**

Supplement: Supplementary file 5 — Additional file 5: Figure S3. Effects of NaCl and PS01 on A. thaliana salt tolerance in vitro under different NaCl concentrations. Non-inoculated A. thaliana grown on MS ½ supplemented with NaCl 0 mM (A1), NaCl 75 mM (A2), NaCl 150 mM (A3),175 mM (A4), 200 mM (A5), 225 mM (A6). A. thaliana inoculated with E. coli grown on MS ½ supplemented with NaCl 0 mM (B1), NaCl 75 mM (B2), NaCl 150 mM (B3), 175 mM (B4), 200 mM (B5), 225 mM (B6). A. thaliana inoculated with PS01 grown on MS ½ supplemented with NaCl 0 mM (C1), NaCl 75 mM (C2), NaCl 150 mM (C3), 175 mM (C4), 200 mM (C5), 225 mM (C6). White bars in the photographs correspond to 1 cm. [file 13104_2019_4046_MOESM5_ESM.docx]
